# Myofibrillar Protein Synthesis Rates Do not Differ With Low and High Estradiol Concentrations Across the Menstrual Cycle

**DOI:** 10.1210/clinem/dgaf410

**Published:** 2025-07-18

**Authors:** Marianna C A Apicella, Tom S O Jameson, Alistair J Monteyne, George F Pavis, Doaa R Abdelrahman, Andrew J Murton, Nima Alamdari, Marlou L Dirks, Benjamin T Wall, Francis B Stephens

**Affiliations:** Nutritional Physiology Research Group, Department of Public Health and Sport Sciences, Faculty of Health and Life Sciences, University of Exeter, Exeter, Devon EX1 2LU, UK; Nutritional Physiology Research Group, Department of Public Health and Sport Sciences, Faculty of Health and Life Sciences, University of Exeter, Exeter, Devon EX1 2LU, UK; Nutritional Physiology Research Group, Department of Public Health and Sport Sciences, Faculty of Health and Life Sciences, University of Exeter, Exeter, Devon EX1 2LU, UK; Nutritional Physiology Research Group, Department of Public Health and Sport Sciences, Faculty of Health and Life Sciences, University of Exeter, Exeter, Devon EX1 2LU, UK; Department of Surgery, University of Texas Medical Branch, Galveston, TX 77555-0177, USA; Sealy Center on Aging, University of Texas Medical Branch, Galveston, TX 77555-0177, USA; Department of Surgery, University of Texas Medical Branch, Galveston, TX 77555-0177, USA; Sealy Center on Aging, University of Texas Medical Branch, Galveston, TX 77555-0177, USA; Nutritional Physiology Research Group, Department of Public Health and Sport Sciences, Faculty of Health and Life Sciences, University of Exeter, Exeter, Devon EX1 2LU, UK; Nutritional Physiology Research Group, Department of Public Health and Sport Sciences, Faculty of Health and Life Sciences, University of Exeter, Exeter, Devon EX1 2LU, UK; Human and Animal Physiology, Wageningen University, 6708 PB Wageningen, The Netherlands; Nutritional Physiology Research Group, Department of Public Health and Sport Sciences, Faculty of Health and Life Sciences, University of Exeter, Exeter, Devon EX1 2LU, UK; Nutritional Physiology Research Group, Department of Public Health and Sport Sciences, Faculty of Health and Life Sciences, University of Exeter, Exeter, Devon EX1 2LU, UK

**Keywords:** estradiol, menstrual cycle, muscle protein synthesis

## Abstract

**Context:**

Skeletal muscle can respond and adapt to sex hormones; however, the degree to which fluctuations in endogenous estradiol across the menstrual cycle (MC) influences rates of myofibrillar protein synthesis (MyoPS) is not clear.

**Objective:**

We compared MyoPS in postabsorptive and postprandial postexercise states, during the early follicular (EF; low estradiol) and late follicular (LF; high estradiol) phases of the MC.

**Methods:**

Seventeen healthy women (age: 28 ± 7 y; body mass index: 24 ± 3 kg.m^2^), participated in a randomized, crossover trial, during the EF (day 4 ± 1; estradiol, 183 ± 78 pmol.L^−1^) and LF (day 15 ± 3; estradiol, 855 ± 571 pmol.L^−1^) phases. Following a resistance exercise bout, participants ingested an amino acid (AA) drink. Blood and muscle samples were collected, pre and post exercise and AA ingestion. Following primed-continuous infusion of L-[*ring*-^2^H_5_]-phenylalanine, MyoPS was measured prior to and during a 4 hour postprandial postexercise period.

**Results:**

MyoPS increased above postabsorptive rates between 0-2 hours to 0.111 ± 0.049 and 0.117 ± 0.058%.h^−1^ (*P* < .001) but not between 2-4 hours (*P* = .522), for EF and LF, respectively, with no interactions observed (*P* = .971). Moderate correlations were shown between total and free testosterone and 0-4 hour MyoPS (r = 0.364, *P* = .048; r = 0.369, *P* = .045, respectively). Expression of several genes associated with protein synthesis, muscle remodeling, and inflammation were increased in LF vs EF (*P* < .050), whereas protein breakdown genes were decreased.

**Conclusion:**

Despite a gene expression profile consistent with muscle growth, MyoPS did not differ with elevated estradiol concentrations. Hence, estradiol does not seem to be important for acutely regulating muscle mass in eumenorrheic women.

The combination of amino acid (AA) ingestion and resistance exercise are essential to maximally stimulate muscle protein synthesis (MPS) and facilitate muscle mass maintenance, reconditioning, and hypertrophy (1). Skeletal muscle is also thought to respond to the internal endocrinological environment. However, the degree to which endogenous fluctuations in circulating sex hormone concentrations following resistance exercise or across the menstrual cycle (MC) can affect muscle protein turnover and, thus, the regulation of muscle mass is not clear (2, 3). Despite this lack of clarity, some studies have recommended MC phase-based resistance exercise training to align with sex hormone fluctuations to optimize MPS, hypertrophy, and performance (4-7), with a particular focus on capturing peaks in circulating estradiol (the most potent form of estrogen in premenopausal females (8)).

Estradiol can bind to nuclear estrogen receptors (ER), ERα, and to a lesser extent ERβ (9), and to membrane bound G protein–coupled receptors within skeletal muscle. Once bound, estradiol has the potential to initiate downstream transcription and signaling cascades such as protein kinase B (AKT) and mitogen-activated protein kinase (10), which are key pathways involved in MPS and muscle hypertrophy (11). In ovariectomized female rats, exogenous estradiol has been shown to reduce muscle damage (12) and increase satellite cell activation through ER-mediated mechanisms (12), and the absence of estradiol via ovariectomy prevents the recovery of atrophied muscle via the AKT pathway (13). However, such models are difficult to translate to humans, and ovariectomy per se has been demonstrated to dramatically increase MPS (14). The most direct evidence on the role of estradiol in human skeletal muscle is derived from studies in postmenopausal women, where estradiol replacement has either not changed (15) or decreased postprandial MPS but increased postresistance exercise rates of MPS (16). Given that exogenous estrogens have differing physiological actions than endogenous estrogens with altered ER binding affinity (17), it is imperative to assess the role of estradiol in vivo using naturally cycling women. This is particularly important as the abundance of ERα in muscle has been shown to change over the course of a MC (18).

To our knowledge, only two studies to date have investigated the effect of MC phase on the MPS response to exercise (19, 20). Both studies reported no differences in rates of exercise-stimulated MPS between the follicular and luteal phases of the MC. However, the role of AA ingestion per se was not assessed, and the circulating concentrations of other sex hormones, particularly progesterone, which is thought to antagonize the effect of estradiol (3, 21), were also different between phases in both studies. Thus, although ecologically valid conclusions can be drawn on the effect of MC phase on MPS, the interaction between the sex hormones makes it difficult to elucidate a specific effect of estradiol. To gain further insight, one experimental approach is to study women more precisely in the early follicular (EF) vs late follicular (LF) phase of the MC where estradiol is low and high, respectively, with minimal changes in progesterone and testosterone, and where luteinizing hormone (LH) and follicle-stimulating hormone (FSH) have not yet peaked (22, 23).

We used multiple physiological techniques to provide a holistic view of the effect of low and high circulating estradiol, during the EF and LF phases of the MC respectively, on skeletal MPS in eumenorrheic women. The primary aim of the present study was to determine the effect of low and high estradiol concentrations, during the EF and LF phase of the MC respectively, on myofibrillar protein synthesis rates (MyoPS) measured in postabsorptive and postprandial postexercise states. An arterialized venous-deep venous (AV-V) balance approach applied across the forearm measured AA uptake from MPS, AA release from muscle protein breakdown (MPB), and balance of all individual AAs and the expression of genes associated with protein turnover, muscle remodeling, and inflammation were measured to provide further insight into the effect of estradiol on skeletal muscle. In addition, we correlated each of the circulating sex hormone concentrations with MyoPS. We hypothesized that elevated estradiol during the LF phase would have an anabolic effect in skeletal muscle.

## Materials and Methods

### Participants

Seventeen healthy (age: 28 ± 7 y; weight: 67 ± 9 kg; body mass index [BMI]: 24 ± 3 kg.m^2^), tier 1 (recreationally active) (24), naturally menstruating and eumenorrheic women, aged 18 to 40 years (White and 1 Asian) volunteered to participate in this randomized, crossover study. Participants' characteristics are displayed in Table 1. Participants had regular MCs, determined by a cycle length between 21 to 35 days and the presence of at least 9 periods in the past year. Information about MC length, frequency, and prevalence of known MC dysfunction was obtained via a MC history questionnaire. To ensure minimal background tracer enrichment, participants had not undergone any stable isotope tracer studies for 6 months prior to enrollment. Exclusion criteria consisted of metabolic impairment, cardiovascular disease, smoking, medication that could affect protein metabolism, MC dysfunction, use of hormonal contraceptives in the 3 months prior to recruitment, pregnancy, and breastfeeding. Participants were admitted into the study when deemed healthy, based on blood pressure (≤140/90 mmHg), BMI (18.5-30 kg.m^2^), and responses to a medical health questionnaire. Experimental procedures, potential risks, and aims of the study were explained to participants prior to obtaining verbal and written informed consent. This study was part of a larger project investigating the effect of MC phase and protein dose on muscle protein synthesis, registered at clinicaltrials.gov (NCT05178732) and approved by the Sport and Health Sciences Ethics Committee of the University of Exeter (21-10-20-B-03), in accordance with the standards for human research as outlined in the Declaration of Helsinki.

**Table 1.** Participants' characteristics

Age, y	28 ± 7
Height, cm	167 ± 7
Weight, kg	67 ± 9
BMI	24 ± 3
Fat, % body mass	27 ± 9
Lean mass, kg	49 ± 6
Leg press 1 RM, kg	83 ± 22
Leg extension 1 RM, kg	28 ± 10
Energy, kcal.day^−1^	1850 ± 399
Protein, g.kg.d^−1^	1.2 ± 0.3
Carbohydrate, g.kg.d^−1^	3.1 ± 0.6
Fat, g.kg.d^−1^	1.2 ± 0.4

Values are mean ± SD.

Abbreviations: BMI, body mass index; RM, repetition maximum.

### Pretesting

After obtaining informed consent, body mass, height, blood pressure, and body composition (using Air Displacement Plethysmography; BodPod, Life Measurement Inc) were measured. Participants then completed unilateral 3 repetition maximum (RM) strength testing on leg press and leg extension machines, using the dominant leg. Given that most of the participants would not be used to regular, maximal unilateral exercise, they were fully familiarized, and the dominant leg was selected for the exercise to obtain the optimal technique and to ensure the maximum weight was lifted. The last weight correctly lifted prior to a failed attempt (± 2.5 kg) was selected as the 3 RM. From the 3 RM, 1 RM was calculated to determine the weight required for the experimental trials. Prior to study completion, participants' habitual dietary intake was assessed via a 3-day weighted food diary, in which 2 weekdays and 1 weekend day were reported. Food diaries were subsequently analyzed for energy and macronutrient intakes (Nutritics Ltd) (see Table 1).

### Familiarization

Participants completed a familiarization visit at least 5 days prior to the first experimental trial. Participants performed a warmup set at 50% of the determined 1 RM for 10 repetitions with the dominant leg. Following the warmup, 4 sets of unilateral exercise at 70% 1 RM on both the leg press and leg extension were completed. Participants were encouraged to work until volitional exhaustion during each set and across both phases of the MC, failure was estimated to occur after 10 repetitions. If more than 12 repetitions were performed, the weight was increased and if fewer than 8 repetitions were completed, the weight was reduced. Participants were provided with 2 minutes of rest in between sets and standardized verbal encouragement was provided throughout.

### Menstrual Cycle Monitoring and Phase Determination

Following the screening visit, participants were asked to track their MCs for 2 complete cycles, as recommended by Elliott-Sale et al (25), to rule out any menstrual dysfunction and determine when the experimental test days took place. Each cycle was calculated starting from the first day of menstrual bleeding until the day before the next bleeding. Participants were asked to report the first day of menstruation and use ovulation kits (Advanced Digital Ovulation Test, Clearblue, Unipath Ltd) until a positive test was attained.

Testing during the EF phase was conducted between the first and fifth day of the MC, to ensure estradiol and progesterone concentrations were both low (25). Testing during the LF phase was conducted when the ovulation kit reported a high result, which was prior to but as close to the LH surge as possible (25, 26); to allow estradiol concentrations to be high and nearing the peak, previous tracking information and ovulation data were used to determine when this phase would occur. The order of these trials was randomized. The gold-standard 3-step method was used to ascertain the phases of the MC, calendar-based counting, urinary LH measurements, and serum hormone analysis (27). Data were included in the analysis only when the LF estradiol concentrations were higher than in the EF phase and when LF progesterone concentrations were less than 16 nmol.L^−1^ (26). Progesterone concentration below 16 nmol.L^−1^ is the recommended acceptable cutoff to ensure low progesterone levels during the LF phase, which are expected to increase by approximately 21-fold when levels peak during the mid-luteal (ML) phase (28).

Following testing during the LF phase, participants were asked to continue using the ovulation kits to determine the LH surge. In line with best practice, around a week following the LH surge (day 22 ± 3; 7 ± 2 days following LH surge), a single blood sample was taken via venipuncture to measure serum progesterone concentrations during the ML phase, to identify if participants were luteal phase deficient (26). Two participants had ML progesterone concentrations below 16 nmol.L^−1^, hence may be luteal phase deficient; however, given we are assessing only the follicular phase, we have included these participants within the analysis.

### Experimental Protocol

**Figure 1.**
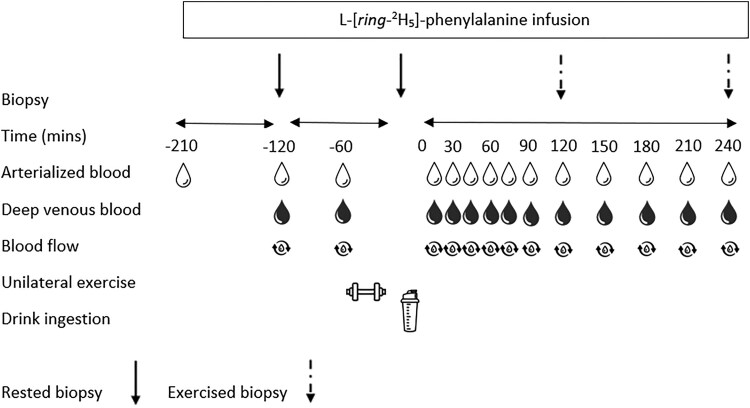
Schematic of the experimental protocol.

An overview of the experimental protocol is shown in Fig. 1. Participants were asked to avoid vigorous physical activity, alcohol, and anti-inflammatory medication in the 48 hours preceding the trial days. All participants were provided with a standardized meal (543 kcal, 16.8 g fat, 60.6 g carbohydrate, 34.0 g protein), to consume by 2000 hours the night before the experimental trial, after which no other food was permitted. The day of the experimental trials, participants arrived at the laboratory at approximately 0800 hours and rested on a bed in a semisupine position. A cannula was inserted antegrade into an antecubital forearm vein, from which a baseline blood sample was collected to measure background tracer enrichment and confirm MC phase via measurement of estradiol and progesterone. The phenylalanine pool was primed with an infusion of 2.94 μmol kg^−1^ L-[*ring*-^2^H_5_]-phenylalanine (*t* = –210 minutes); thereafter, a continuous infusion was maintained at a rate of 0.049 μmol·kg^−1^·min^−1^ for the duration of the test day. A second cannula was inserted retrograde into a dorsal hand vein of the same arm, and placed into a heated hand warmer (55 °C), for collection of repeated arterialized venous blood samples (29). A third cannula was placed retrograde into a deep-lying forearm vein to sample blood from the forearm muscle bed (30, 31). Simultaneous AV-V blood samples were collected throughout the experimental day at the following time points: *t* = −120, −60, 15, 30, 45, 60, 75, 90, 120, 150, 180, 210, and 240 minutes. Blood flow of the brachial artery was determined via ultrasound imaging immediately prior to every blood sample. Luminal diameter was measured for at least 2 seconds, 5 cm proximal to the antecubital fossa (31). From the same anatomical position, mean blood velocity was measured for a minimum of 8 cardiac cycles (32). Files were analyzed using Brachial Analyzer for Research, version 6 (Medical Imaging Applications LLC). After 120 minutes from the start of the infusion, a baseline muscle biopsy was collected from the nondominant, rested leg. Muscle biopsies were taken from the middle region of the m. vastus lateralis using a modified Bergström needle, under local anesthesia using 2% lidocaine. All biopsy samples were freed from any visible blood, adipose, and connective tissue, frozen in liquid nitrogen, and stored at—80 °C until subsequent analysis. At *t* = −60 minutes participants were transferred to the research gym via wheelchair, to ensure minimal weight was applied to the rested leg, to complete the 30-minute unilateral resistance exercise, as described previously (see “Familiarization”). On completion of the exercise protocol (120 minutes after the initial biopsy), a second biopsy was taken from the rested leg, 1 to 2 cm proximal from the previous incision. Immediately following this biopsy (*t* = 0), participants consumed an AA beverage, (see “Experimental beverage”), within an allocated 5-minute period. After the drink was consumed, this initiated a 240-minute (4-hour) postprandial postexercise period during which participants rested in a semi-supine position. During this postprandial postexercise period, muscle biopsies were collected from the exercised leg at *t* = 120 and 240 minutes to assess rates of MyoPS and gene expression (240 minutes only).

### Experimental Beverage

This study was part of a larger study investigating the effect of MC phase and leucine dose (0.6 g, 1.5 g, and 2.0 g of leucine) on MyoPS. Data from this larger study were pooled to assess the effect of estradiol per se on MyoPS in the present study. Details of drink preparation have been published previously (33); of note, drinks were enriched with 2% L-[*ring*-^2^H_5_]-phenylalanine to minimize changes in blood isotopic enrichment following ingestion of nonlabeled phenylalanine (34). Following the consumption of the drinks, 100 mL of water was added to wash the bottle and ensure all drink was consumed. The same drink was provided during both phases of the MC, and postexercise MyoPS has been shown to not differ between these drink conditions (33). The nutritional content of the experimental drinks is displayed in Table 2.

**Table 2.** Nutritional content of the experimental drinks

	1.5 g EAA	15 g Whey protein	20 g Whey protein
**Macronutrients**			
Protein, g	1.2	15.4	19.5
Carbohydrate, g	4.2	0.9	1.1
Fat, g	0.1	1.0	1.3
Fiber, g	0.5	1.2	1.5
Energy, kcal	23.9	76.8	96.0
Energy, kJ	101.3	324.6	405.8
**Amino acid content, g**			
Alanine	<0.001	0.85	1.06
Arginine	0.01	0.38	0.48
Aspartic acid	0.01	1.90	2.37
Cystine	<0.001	0.37	0.46
Glutamic acid	0.02	2.78	3.48
Glycine	<0.001	0.31	0.39
Histidine	0.02	0.27	0.34
Isoleucine	0.17	0.75	0.94
Leucine	0.59	1.54	1.95
Lysine	0.24	1.58	1.97
Methionine	0.05	0.33	0.42
Phenylalanine	0.08	0.47	0.58
Proline	<0.001	0.94	1.17
Serine	0.01	0.95	1.18
Threonine	0.19	1.09	1.36
Tryptophan	0.01	0.31	0.39
Tyrosine	<0.001	0.48	0.60
Valine	0.18	0.71	0.89
**Sum of amino acids, g**			
TAA	1.57	16.0	20.0
EAA	1.5	7.4	9.3
BCAA	0.9	3.0	3.8
**Polyphenol content (GAE/serving)**			
Polyphenol content	252.2	n/a	n/a

The experimental drinks contained 6.4, 20, and 25 g of total product for 1.5, 15, and 20 g, respectively.

Abbreviations: BCAA, branched-chain amino acids; EAA, essential amino acids; GAE, gallic acid equivalent; TAA, total amino acids.

### Blood Sample Collection and Analysis

A total of 8 mL of AV-V blood samples were collected into a syringe at each time point. A total of 5 mL of each sample were added to lithium-heparin tubes (BD vacutainer LH; BD Diagnostics), centrifuged immediately at 4000 rpm at 4 °C for 10 minutes, with the plasma supernatant aliquoted and stored at −80 °C until analysis. The remaining 3 mL were added to serum separator tubes (BD vacutainers SST II, BD Diagnostics), left to clot at room temperature for 30 minutes before being centrifuged at 4000 rpm at 4 °C for 10 minutes. The serum supernatant was then removed, aliquoted, and stored at −80 °C until analysis.

Serum estradiol, FSH, LH, progesterone, sex hormone–binding globulin (SHBG), and total testosterone were determined via electrochemiluminescence using the Cobas e 801 automated analyzer (Roche Diagnostics), with albumin determined using the Cobas c 702 (Roche Diagnostics). Serum free testosterone was calculated using total testosterone, albumin, and SHBG (35). Free estradiol index was calculated as serum estradiol divided by SHBG. Estradiol to progesterone ratio was calculated by converting estradiol from pmol.L^−1^ to nmol.L^−1^, then dividing by progesterone (nmol.L^−1^). Plasma glucose was measured using a YSI glucose analyzer (YSI 2500). Serum insulin concentrations were measured using a commercially available enzyme-linked immunosorbent assay kit (DRG Insulin ELISA, EIA-2935, DRG International Inc, RRID:AB_2891339).

Plasma L-[*ring*-^2^H_5_]-phenylalanine enrichments and concentrations of alanine, glutamic acid, histidine isoleucine, leucine, lysine, methionine, phenylalanine, proline, serine, threonine, tyrosine, and valine were determined by gas chromatography-mass spectrometry (GC-MS), as described previously (34). Briefly, to prepare samples for GC-MS, 10 μL of 2 mM norleucine was added as an internal standard to 500 μL of plasma and deproteinized on ice with 500 μL of 15% 5-sulfosalcylic acid. Samples were vortexed and centrifuged at 4000*g* for 10 minutes at 4 °C, with the supernatant loaded onto cation-exchange columns. Columns were filled with ddH_2_O, 6 mL 0.5 M acetic acid, and washed 5 times with ddH_2_O. The AAs were then eluted from the columns with 2 mL of 6 M ammonium hydroxide and dried using a Speed-Vac for 8 hours at 60 °C prior to derivatization and analysis by GC-MS.

### Skeletal Muscle Tissue Analysis

Muscle biopsy samples were analyzed for MyoPS based on the incorporation of L-[*ring*-^2^H_5_]-phenylalanine. The myofibrillar fraction was extracted, as previously described (34). Briefly, approximately 50 mg muscle tissue was homogenized and centrifuged, and the sarcoplasmic supernatant was removed. The remaining pellet was solubilized in 0.3 M sodium hydroxide, centrifuged, and separated from the collagen pellet. The remaining myofibrillar proteins were precipitated with the addition of 1 M perchloric acid, washed twice in 70% ethanol, and hydrolyzed in 6 M hydrochloric acid at 110 °C for 24 hours. The AAs were purified using cation-exchange columns and dried using a Speed-Vac for 8 hours at 60 °C. Following derivatization, the myofibrillar protein-bound L-[*ring*-^2^H_5_]-phenylalanine enrichments were determined using GC-MS, as previously described (34).

Total RNA was extracted from approximately 20 mg skeletal muscle using TRIzol reagent, following the method of Chomczynski and Sacchi (36) from muscle collected from the first (postabsorptive) and final (4 hour postprandial postexercise) biopsy. RNA was spectrophotometrically quantified at 260 nm, and purity was determined as the ratio of readings at 260/280 nm (NanoDrop Lite Spectrophotometer, Thermo Fisher Scientific). First-strand complementary DNA was synthesized from RNA using a commercially available kit (SuperScript III First-Strand Synthesis SuperMix, Thermo Fisher Scientific). TaqMan low-density custom array cards (Thermo Fisher Scientific) were used for the quantification of the expression of genes associated with protein synthesis, protein breakdown, muscle remodeling, and inflammation. Prior to running the cards, 50 μL of TaqMan Universal Master Mix II (Thermo Fisher Scientific) and 5 μL of RNAse free water was added to 50 μL complementary DNA (150 ng of RNA equivalent). Samples were vortexed, centrifuged, and 100 μL of sample was loaded onto each card reservoir. Cards were then centrifuged and run on a Quantstudio 12 K Flex Fast Real-Time PCR system (Thermo Fisher Scientific). Relative quantification of the genes of interest was performed using the Δ-Δ Ct method. The fold change in messenger RNA (mRNA) levels were expressed relative to eukaryotic 18S ribosomal RNA and a median participant for normalization purposes. A total of 28 genes were included in the final analysis (Supplementary Table S1) (37).

### Calculations

To determine MyoPS, the fractional synthetic rate (FSR) of myofibrillar muscle proteins were calculated using the standard precursor-product equation, equation (1) (38):


(1) $$\mathrm{FSR}\;(\text{\%}h^{\text{-}1})=(\Delta E_{p}/E_{\mathrm{precursor}}x\;t)\;x\;100$$

Where ΔE_p_ is the increase in L-[*ring*-^2^H_5_]-phenylalanine enrichment in myofibrillar muscle protein between two biopsies. E_precursor_ is the weighted average L-[*ring*-^2^H_5_]-phenylalanine enrichment in the plasma over time and *t* is the tracer incorporation time (h) between two muscle biopsies.

Two pool AV-V AA kinetics across the forearm were calculated according to the principles and calculations outlined within Wolfe and Chinkes (2004) (38). As a phenylalanine stable isotope tracer was used, which is not metabolized or synthesized in skeletal muscle, rate of disappearance (R_d_) represents AA uptake from the plasma and can often be used as a proxy for MPS and rate of appearance (R_a_) reflects the appearance of phenylalanine into the plasma, resulting from MPB.

Net balance of phenylalanine was calculated according to the Fick principle, equation (2):


(2) $$\mathrm{NB}=\mathrm{BF}\;x\;(C_{A}\text{–}C_{V})$$

Where NB is the net balance of an AA, BF is blood flow, C_A_ is arterialized venous concentration, and C_V_ is the deep venous concentration of the AA.

Equation (3) was used to calculate AA rates of disappearance, representing uptake from the plasma and into the forearm for MPS:


(3) $$R_{d}=\mathrm{tracer}\;\mathrm{uptake}/E_{A}=([C_{A}xE_{A}\text{-}C_{V}x\;E_{V}]\;x\;\mathrm{BF})/\;E_{A}$$

Where R_d_ is the rate of disappearance of phenylalanine from the plasma, E_A_ is the enrichment of labeled phenylalanine from the arterialized venous blood, E_V_ is the enrichment of labeled phenylalanine from the deep venous blood, C_A_ is arterialized venous concentration, and C_V_ is the deep venous concentration of phenylalanine and BF is blood flow.

Phenylalanine rates of appearance, representing release from the forearm into the plasma from MPB, can be calculated using equation (4):


(4) $$R_{a}=R_{d}\text{-}\;NB_{\mathrm{phe}}$$

Where R_a_ is the rate of appearance of phenylalanine in the plasma and NB_phe_ is the net balance of phenylalanine.

### Statistics

A 2-sided power analysis with a medium effect size revealed n = 14 was sufficient to detect differences in MyoPS between MC phases, when using a repeated-measure analysis of variance (ANOVA) (*P* < .05; power 90%, *f* = 0.4; G*power version 3.1.9.7, RRID:SCR_013726), given that estradiol supplementation has been shown to increase MyoPS in response to resistance exercise by 30% (16). To account for a 20% dropout rate and a post hoc exclusion rate of up to approximately 40% (26), 23 participants were recruited for the study, with 6 dropouts or exclusions (personal reasons, n = 2; unpleasant biopsy, n = 2; irregular MC, n = 1; and oligomenorrhea, n = 1), therefore data are presented for n = 17.

SHBG and exercise volume between MC phases were analyzed using a paired *t* test. Data were not normally distributed for estradiol, estradiol to progesterone ratio, free estradiol index, free testosterone, FSH, LH, progesterone, total testosterone, and background L-[*ring*-^2^H_5_]-phenylalanine enrichments between MC phases, therefore data were analyzed by the Wilcoxon signed-rank test. Differences in plasma glucose concentrations, serum insulin concentrations, blood flow, plasma AA concentrations, AA balance, rates of disappearance of phenylalanine, rates of appearance of phenylalanine, plasma-[*ring*-^2^H_5_]-phenylalanine enrichments, myofibrillar L-[*ring*-^2^H_5_]-phenylalanine enrichments, myofibrillar FSR, and muscle gene expression between the 2 phases of the MC were analyzed using a 2-way repeated-measure ANOVA (time × phase), with time and phase (levels: EF and LF) as independent factors. When data were not normally distributed, these were log-transformed prior to analysis. When statistically significant interactions were observed, Sidak post hoc tests were performed to identify individual differences. Total postprandial insulin availability was calculated as incremental area under the curve with baseline set as an average of *t* = −120 and −60 minutes and analyzed using a paired *t* test. The relationship between SHBG and myofibrillar FSR was assessed using Pearson product moment correlation and, for the remainder of the sex hormones that were not normally distributed, relationships were analyzed using Spearman rank correlation. Where significant correlations were observed, partial correlations were run, while controlling for some of the measured sex hormones as covariates. Statistical analyses were performed using GraphPad Prism version 10.4.1 (RRID:SCR_002798) and SPSS statistics version 28.0.1.1 (IBM, RRID:SCR_016479). Statistical significance was set to *P* < .050. Data are expressed as mean ± SD.

## Results

### Hormone Concentrations

**Figure 2.**
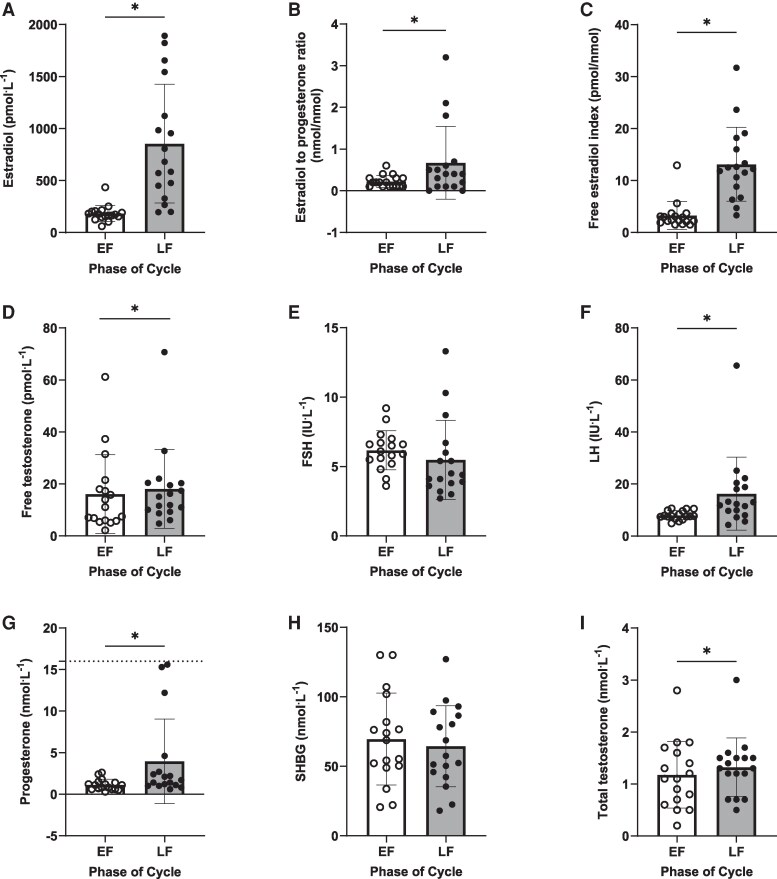
Individual circulating concentrations of A, estradiol; B, estradiol to progesterone ratio; C, free estradiol index; D, free testosterone; E, follicle-stimulating hormone (FSH); F, luteinizing hormone (LH); G, progesterone; H, sex hormone–binding globulin (SHBG); and I, total testosterone across the early follicular (EF) and late follicular (LF) phases of the menstrual cycle in healthy young women (n = 17). The dashed line on G represents 16 nmol.L^−1^, the preestablished upper limit of inclusion for progesterone concentrations during the LF phase. Data were analyzed using paired samples *t* test, and when data were not normally distributed the Wilcoxon signed rank test was used. Values are mean ± SD. *A statistically significant difference between phases.

**Figure 3.**
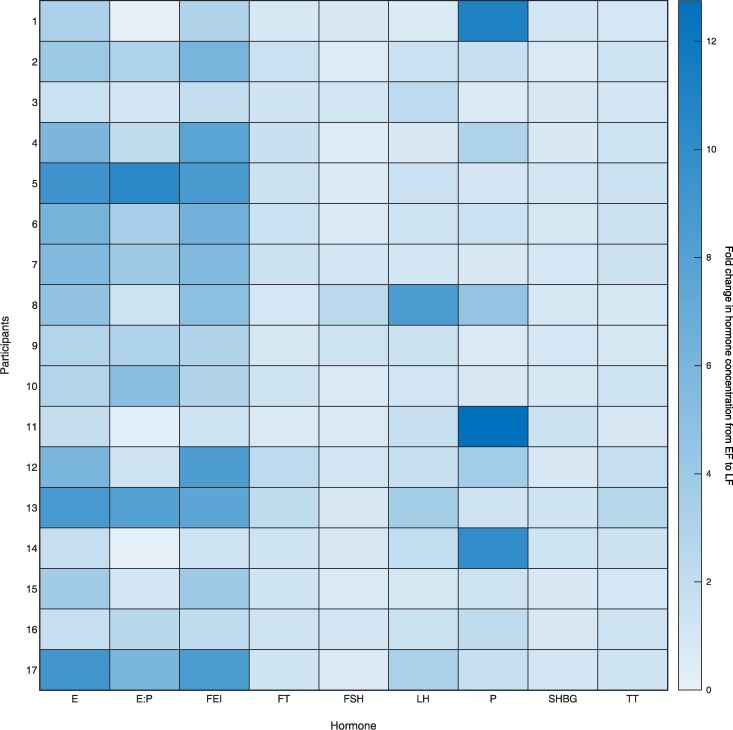
Circulating concentrations of estradiol (E), estradiol to progesterone ratio (E:P), free estradiol index (FEI), free testosterone (FT), follicle-stimulating hormone (FSH), luteinizing hormone (LH), progesterone (P), sex hormone–binding globulin (SHBG), and total testosterone (TT) in healthy young women (n = 17), displayed as a heat map. Data are presented as the fold change from the early follicular (EF) to late follicular (LF) phase with lighter shades representing a small fold change and darker shades representing a large fold change between phases.

Serum estradiol (855 ± 571 vs 183 ± 78 pmol.L^−1^), free estradiol index (13 ± 7 vs 3 ± 3 pmol/nmol), and estradiol to progesterone ratio (0.7 ± 0.9 vs 0.2 ± 0.1 nmol/nmol) were all markedly greater in LF compared to EF (all *P* < .050) (Figs. 2 and 3). LH, progesterone, total testosterone, and free testosterone were also elevated in LF compared to EF, albeit to a lesser degree, and only in some participants (see Fig. 3), whereas no differences were observed for FSH and SHBG between phases (all *P* > .050) (see Figs. 2 and 3).

### Total Work

Total work (repetition × weight) executed across both leg press and leg extension exercises was trending to be greater during the LF compared to the EF phase (3573 ± 1047 vs 3361 ± 933 kg; *P* = .065).

### Plasma Glucose and Serum Insulin Concentrations

**Figure 4.**
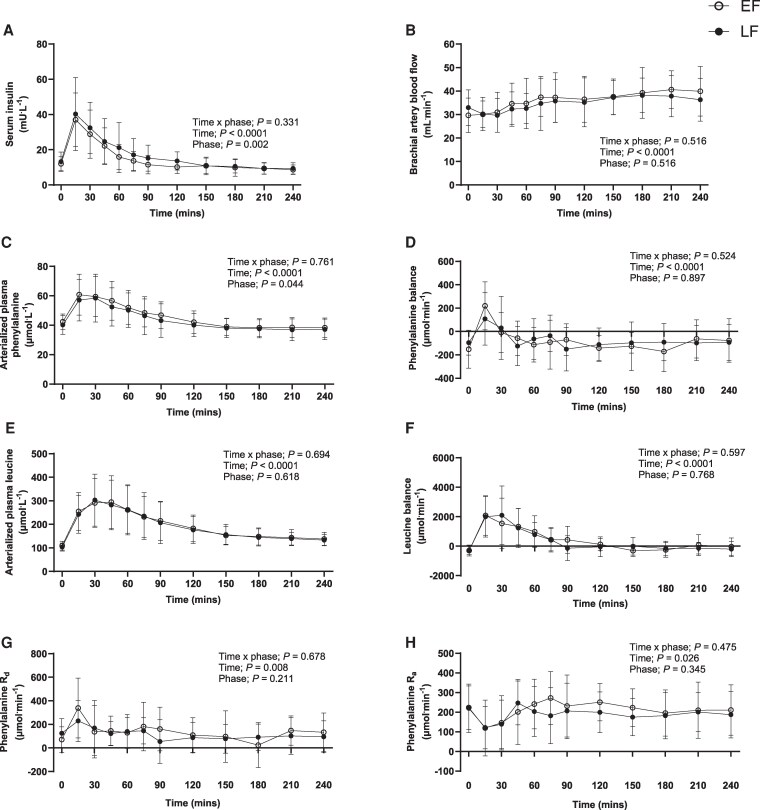
Time course of A, serum insulin; B, brachial artery blood flow; C, arterialized plasma phenylalanine; D, phenylalanine forearm balance; E, arterialized plasma leucine; F, leucine forearm balance; G, forearm phenylalanine R_d_ (representative of muscle protein synthesis); and H, forearm phenylalanine R_a_ (representative of muscle protein breakdown) over the average of the postabsorptive period (time point 0) and a 4-hour postprandial period in healthy young women (n = 17, for insulin, phenylalanine, and leucine concentrations; n = 14, for blood flow; n = 13, for the rest). Data were analyzed using a 2-way repeated-measure analysis of variance (time × phase) with Sidak post hoc tests used to detect differences at individual time points. Values are mean ± SD.

Plasma glucose concentrations (Supplementary Fig. S1 (37)) decreased below postabsorptive values during the postprandial postexercise period from *t* = 45 to 60 minutes and from *t* = 120 to 240 minutes (time effect; *P* < .0001), with no differences between phases (time × phase interaction; *P* = .503). Postabsorptive insulin concentrations did not differ between phases (EF, 12 ± 3 vs LF, 14 ± 4 mU·L^−1^; *P* = .085). During the postprandial postexercise period, serum insulin concentrations (Fig. 4A) increased above postabsorptive values from 15 to 45 minutes (time effect; *P* < .0001), this was not different between phases over time (time × phase interaction; *P* = .331). Serum insulin was greater overall in LF compared to EF (phase effect; *P* = .002) but postprandial serum insulin incremental area under the curve was not different between phases (*P* = .419; data not shown).

### Brachial Artery Blood Flow

Cannulation of the deep vein was not possible for 3 participants; data are therefore presented for 14 participants. Postabsorptive brachial artery blood flow was higher during LF in comparison to EF (33 ± 8 vs 30 ± 7 mL·min^−1^; *P* = .046). Brachial artery blood flow (Fig. 4B) increased above postabsorptive values from *t* = 150 onward (time effect; *P* < .0001), with no differences between phases over time (time × phase interaction; *P* = .516).

### Plasma Amino Acid Concentrations and Kinetics

As cannulation of the deep vein was not possible for 3 participants and not possible for 1 visit of another participant, phenylalanine and leucine forearm balance and forearm phenylalanine R_d_ and R_a_ are presented for 13 participants. Arterialized plasma phenylalanine (Fig. 4C) and leucine (Fig. 4E) concentrations increased over the postprandial postexercise period (time effect; both *P* < .0001), with no differences between phases over time (time × phase interaction; *P* = .761; *P* = .694, respectively). Plasma phenylalanine concentrations were increased above postabsorptive values from *t* = 15 to 60 minutes, and plasma leucine was increased above postabsorptive values from *t* = 15 to 150 minutes. Arterialized plasma concentrations of all remaining measured AAs can be found in Supplementary Fig. S2 (37).

During the postprandial period, phenylalanine (Fig. 4D) and leucine (Fig. 4F) forearm balance increased from negative to positive (time effect; both *P* < .0001), with no differences between phases over time (time × phase interaction; *P* = .524; *P* = .597, respectively). Forearm phenylalanine balance was increased above postabsorptive values at *t* = 15 minutes, and leucine balance was increased above postabsorptive values from *t* = 15 to 60 minutes. Forearm phenylalanine R_d_ (Fig. 4G) and R_a_ (Fig. 4H) increased and decreased, respectively, compared to postabsorptive values at *t* = 15 minutes (time effect; *P* = .008; *P* = .026, respectively), with no differences between phases (time × phase interaction; *P* = .678; *P* = .475, respectively). Arterialized plasma balance of all remaining measured AAs can be found in Supplementary Fig. S3 (37).

### Plasma and Skeletal Muscle Tracer Analysis

Plasma L-[*ring*-^2^H_5_]-phenylalanine enrichments (Supplementary Fig. S4 (37)) changed over time (time effect; *P* < .0001), with no differences between phases (time × phase interaction; *P* = .085). Plasma L-[*ring*-^2^H_5_]-phenylalanine enrichments decreased transiently below postabsorptive values between 15 and 30 minutes and increased above postabsorptive values between 150 and 240 minutes.

Due to insufficient muscle tissue for 2 participants, data for muscle L-[*ring*-^2^H_5_]-phenylalanine are presented for 15 participants. Myofibrillar protein-bound L-[*ring*-^2^H_5_]-phenylalanine enrichments did not differ between phases at baseline (*P* = .902). Myofibrillar protein-bound L-[*ring*-^2^H_5_]-phenylalanine enrichments increased, with no differences between phases (time effect; *P* < .0001; time × phase interaction; *P* = .478) during the postabsorptive period, in EF from 0.033 ± 0.026 to 0.041 ± 0.025 mole percent excess (MPE) and in LF from 0.039 ± 0.018 to 0.047 ± 0.017 MPE.

In a postprandial postexercise state, myofibrillar protein-bound L-[*ring*-^2^H_5_]-phenylalanine enrichments increased over time and with no differences between phases (time effect; *P* < .0001; time × phase interaction; *P* = .643). Myofibrillar protein-bound L-[*ring*-^2^H_5_]-phenylalanine enrichments increased from 0.041 ± 0.025 to 0.058 ± 0.028 then 0.068 ± 0.028 MPE and from 0.047 ± 0.017 to 0.065 ± 0.022 then 0.086 ± 0.037 MPE, from 0 hours, 2 hours, and 4 hours during the postprandial postexercise period for EF and LF, respectively.

**Figure 5.**
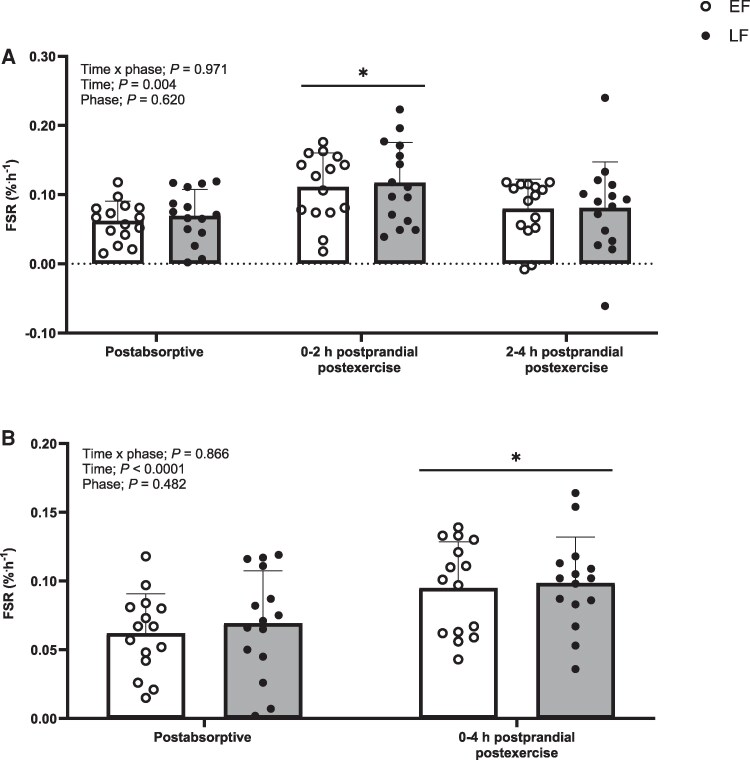
Myofibrillar protein fractional synthetic rates (FSR) calculated using the plasma L-[*ring*-^2^H_5_]-phenylalanine precursor pool for A, a postabsorptive and postprandial postexercise periods (0-2 hours and 2-4 hours) and B, a total 4 hour postprandial postexercise period during the early follicular (EF) and late follicular (LF) phases of the menstrual cycle in young healthy women (n = 15). Data were analyzed using a 2-way repeated-measure analysis of variance (time × phase) with Sidak post hoc tests used to detect differences at individual time points. Values are mean ± SD. *A statistically significant difference between postabsorptive and postprandial postexercise conditions.

Postabsorptive FSRs did not differ between phases (EF, 0.062 ± 0.029%.h^−1^; LF, 0.069 ± 0.038%.h^−1^; *P* = .580). Myofibrillar FSRs increased above postabsorptive rates between 0-2 hours of the postprandial postexercise period (EF, 0.111 ± 0.049%.h^−1^; LF, 0.117 ± 0.058%.h^−1^; *P* < .001) but not between 2-4 hours (EF, 0.080 ± 0.043%.h^−1^; LF, 0.081 ± 0.066%.h^−1^; *P* = .522), with no differences between MC phases observed at either time point (time × phase interaction; *P* = .971) (Fig. 5A). Accordingly, there were no differences between phases in myofibrillar FSRs over the entire 0-4 -hour postprandial postexercise window (EF, 0.095 ± 0.033%.h^−1^; LF, 0.098 ± 0.033%.h^−1^; time × phase interaction; *P* = .866) (Fig. 5B).

**Figure 6.**
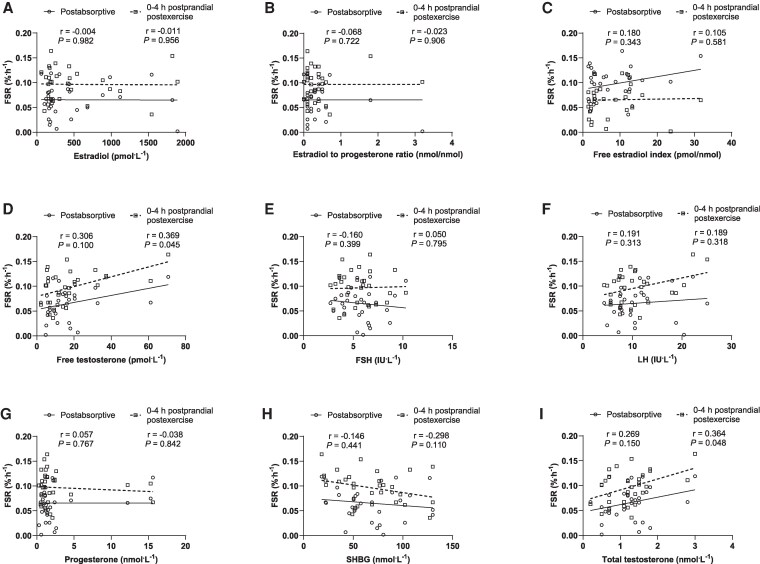
Correlations between myofibrillar protein fractional synthetic rates (FSR) and A, estradiol; B, estradiol to progesterone ratio; C, free estradiol index; D, free testosterone; E, follicle-stimulating hormone (FSH); F, luteinizing hormone (LH); G, progesterone; H, sex hormone–binding globulin (SHBG); and I, total testosterone in a postabsorptive and 0-4 hour postprandial postexercise state in young healthy women (n = 15). Data were analyzed by Pearson correlation analysis, and when data was not normally distributed the Spearman rank test was used.

Speakman rank correlation coefficient showed a moderate correlation between free testosterone and myofibrillar FSR between 0-4 hours (r = 0.369; *P* = .045) (Fig. 6D). In addition, total testosterone moderately correlated with myofibrillar FSRs between 0-4 hours (r = 0.364; *P* = .048) (Fig. 6I). The remainder of the sex hormones (estradiol, estradiol to progesterone ratio, free estradiol index, FSH, LH, progesterone; and SHBG) did not correlate with myofibrillar FSR at any time point (*P* > .050) (see Fig. 6).

To provide greater insight into the correlation between total testosterone and 0-4-hour myofibrillar FSR, partial correlations were run with the remainder of the sex hormones as covariates in the analysis (Supplementary Table S2 (37)). In model 1, results were adjusted for the main hormones of interest, estradiol and progesterone, where a statistically significant correlation remained (r = 0.428; *P* = .023). In model 2, additional adjustments were made for hormones related to estradiol and progesterone: estradiol to progesterone ratio, free estradiol index, and SHBG. The addition of these hormones resulted in minimal change to the correlation (r = 0.414; *P* = .040). Additional adjustments in model 3 for the remaining hormones, FSH and LH, changed the correlation to no longer significant (r = 0.350; *P* = .102).

### Skeletal Muscle Transcriptional Response to Nutrition and Exercise During the Early Follicular and Late Follicular Phases

**Figure 7.**
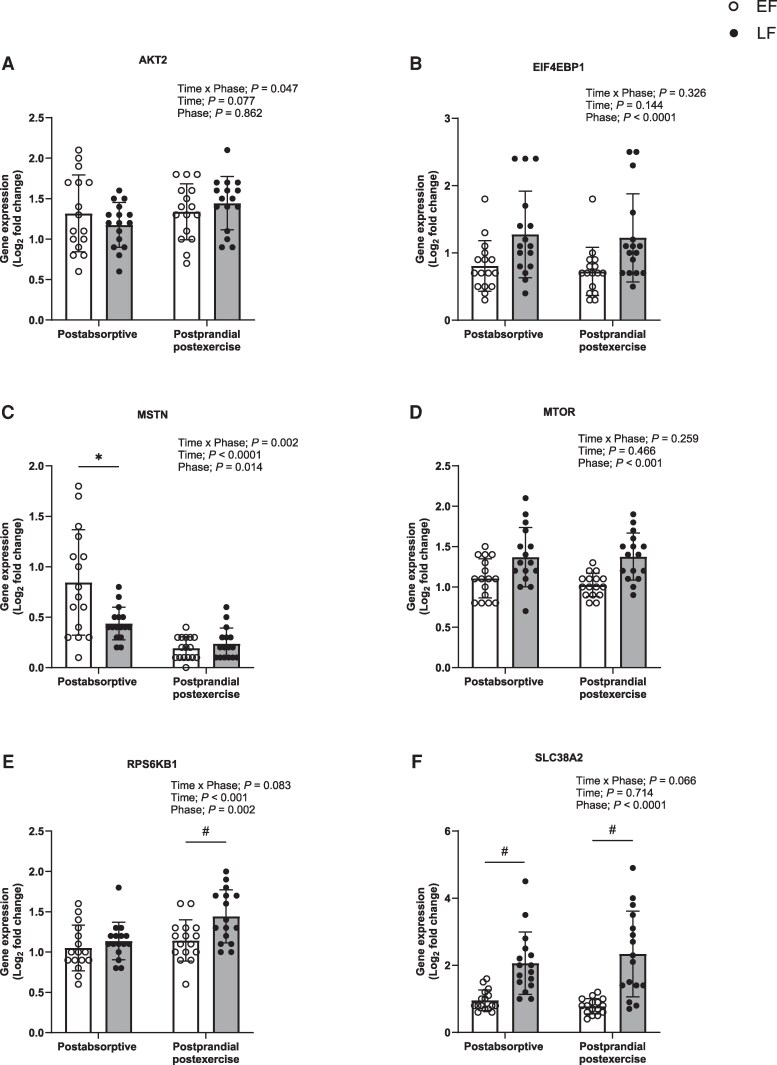
Skeletal muscle expression of genes associated with protein synthesis, A, AKT2; B, EIF4EBP1; C, MSTN; D, MTOR; E, RPS6KB1; and F, SLC38A2 in the early follicular (EF) and late follicular (LF) phases of the menstrual cycle at 2 time points, postabsorptive (−120 minutes) and postprandial postexercise (240 minutes) in young women (n = 16). Data were analyzed using a 2-way repeated-measure analysis of variance (time × phase) with Sidak post hoc tests used to detect differences at individual time points. Values are mean ± SD. *Individual differences between phases at that time point. #A trend for a statistically significant difference between phases at that time point.

**Figure 8.**
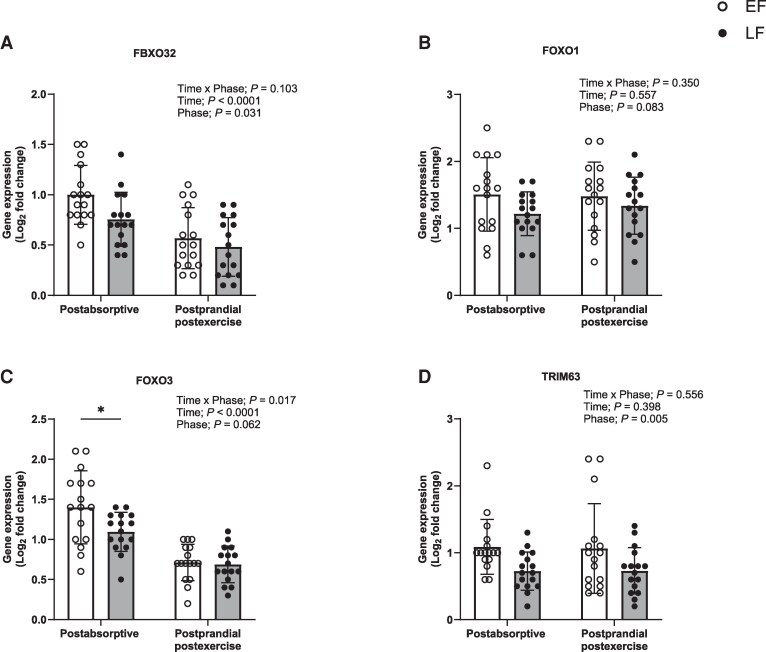
Skeletal muscle expression of genes associated with protein breakdown, A, FBXO32; B, FOXO1; C, FOXO3; and D, TRIM63 in the early follicular (EF) and late follicular (LF) phases of the menstrual cycle at 2 time points, postabsorptive (−120 minutes) and postprandial postexercise (240 minutes) in young women (n = 16). Data were analyzed using a 2-way repeated-measure analysis of variance (time × phase) with Sidak post hoc tests used to detect differences at individual time points. Values are mean ± SD. *Individual differences between phases at that time point.

For mechanistic insight into the effect of estradiol on skeletal muscle, skeletal muscle expression of genes associated with protein synthesis, protein breakdown, muscle remodeling, and inflammation (Figs. 7 and 8 and Supplementary Figs. S5 and S6 (37), respectively) were measured in a postabsorptive and 4-hour postprandial postexercise state. Gene expression data are presented for 16 participants, due to insufficient muscle tissue for 1 participant. As 45% of ESR2 mRNA samples did not amplify, the remaining samples were subsequently removed from any analysis. Seventeen genes exhibited a time effect: mRNA expression either increased (HSP90AA1, HSPB1, HSPB8, IL18, IL1R1, JUN, MYOD1, NFKB1, RPS6KB1, SMAD3, SOD1, and TGFB1) or decreased (ESR1, FBXO32, FOXO3, MTSN, and NFKBIA) in postprandial postexercise muscle (*P* < .050). Nineteen genes displayed a phase effect: mRNA expression either increased (CCND1, EIF4EBP1, HSP90AA1, HSPB1, IL18, IL1R1, MTOR, MYOD1, NKFB1, NFKBIA, PAX7, RPS6KB1, SLC38A2, SOD1, and SOD2) or decreased (FBXO32, MTSN, SMAD3, and TRIM63) (*P* < .050) during LF. There was a tendency for ESR1 to be increased (*P* = .068) and for FOXO1 and FOXO3 to be decreased during the LF phase (*P* = .083; *P* = .062, respectively). Seven genes demonstrated a time × phase interaction (*P* < .050), with divergent responses between phases in either postabsorptive or postprandial postexercise states. mRNA expression of IL18, MYOD1, RELA, and SOD1 increased in postprandial postexercise muscle during LF (*P* < .050), and mRNA expression for IL18 was increased and FOXO3 and MTSN were decreased in postabsorptive muscle during LF. AKT2 showed a time × phase interaction (*P* < .050) but mRNA expression did not differ between phases at any specific time point.

## Discussion

The primary aim of the present study was to determine the effect of low and high estradiol concentrations, during the EF and LF phase of the MC respectively, on MyoPS measured in postabsorptive and postprandial postexercise states. Contrary to our hypothesis, we found no difference in MyoPS between EF and LF phases in a postabsorptive or postprandial postexercise state. In support of our MyoPS data, there was no difference in forearm AA balance (of all AAs measured), uptake from MPS (R_d_ of phenylalanine), or release from MPB (R_a_ of phenylalanine) between low and high estradiol concentrations. MyoPS did not significantly correlate with circulating estradiol, despite greater muscle expression of genes associated with protein synthesis and lower expression of genes associated with protein breakdown during the LF phase, suggestive of a more anabolic environment with higher circulating estradiol. Interestingly, postprandial postexercise MyoPS correlated with both total and free circulating testosterone, suggesting that testosterone may be more important than estradiol for regulating MyoPS in women.

We aimed to study participants in the EF vs LF phase of the MC where estradiol is low and high respectively, with minimal changes in progesterone and testosterone and where LH and FSH have not yet peaked (22, 23). As intended, estradiol concentrations were significantly elevated by around 5-fold in LF compared to EF (855 vs 183 pmol.L^−1^, respectively), which is in line with values measured before for these phases (730 vs 100 pmol.L^−1^) (28), but are above those reported during the LF phase when MPS has been previously assessed (averaging ∼428 pmol.L^−1^) (20). However, despite using the gold-standard 3-step method to validate MC phases (calendar-based counting, urinary LH measurements via ovulation kits, and serum hormone analysis) (27) and employing a randomized, crossover design, there was considerable variation in estradiol concentration between participants in the LF phase, perhaps because not all individuals have a large peak at this time (28). Moreover, whereas FSH did not differ between the two phases, progesterone, testosterone, and LH concentrations were also elevated during the LF phase, albeit not to the same magnitude as estradiol (exemplified in Fig. 3 heat map). The measured concentrations of progesterone, testosterone, and LH were not close to the peak concentrations expected from the literature of around 40 nmol.L^−1^, 1.7 nmol.L^−1^, and 42 IU.L^−1^, respectively (22, 28), and progesterone was still within the acceptable range for this phase (26). Hence, we feel that our model to study estradiol is effective, and we have overcome the limitations with previous work in this area, where MyoPS was measured when multiple hormones were near maximal concentrations while achieving an applied and ecologically valid study (without blocking or supplementing hormones). It is worth mentioning that we did not measure inhibin B, which peaks towards the end of the follicular phase and negatively regulates FSH (39). However, as far as we are aware there is no evidence to suggest either an anabolic or catabolic effect of inhibin B per se on skeletal muscle. The large interindividual variability in estradiol along with the elevated concentrations of the other sex hormones make interpretation of the role of endogenous estradiol challenging. Nevertheless, we observed a significant increase in serum insulin and blood flow during the LF phase, which agrees with previous observations (40, 41) and are both associated with increased estradiol per se (40, 42), so we are confident that our model can provide further insight into the role of endogenous estradiol.

The primary finding from this study is that MyoPS did not differ with low and high estradiol concentrations during the EF and LF phases of the MC in a postabsorptive and postprandial postexercise state. This finding is consistent with that of Miller et al (19), who found no significant difference in MyoPS between EF and ML phases in 15 women following nutrient drink consumption 24 hours after resistance exercise, and Colenso-Semple et al (20), who found no difference in integrated MyoPS over 6 days of the follicular and luteal phases in rested and exercise muscle. The findings are also in agreement with those of Smith et al (15), who reported that postabsorptive MPS rates did not differ in postmenopausal women with or without estradiol supplementation. Thus, taken together, and given that we did not see anticipated maximal concentrations of progesterone and other sex hormones across the MC, this shows that estradiol does not affect MyoPS following AA ingestion and resistance exercise, the main drivers of MPS, and that recommendations on MC phase-based resistance training due to fluctuating estradiol are misplaced. Indeed, we found no correlation between estradiol and MyoPS across a wide range of estradiol concentrations in the women in the present study. On the other hand, it was interesting to note that total and free testosterone, which increase slightly prior to ovulation as a precursor for endogenous estrogen synthesis (23), positively correlated with 0-4 hours postprandial postexercise MyoPS, even after factoring in other sex hormones as covariates. In addition to the clear role of exogenous testosterone stimulating MyoPS both in men and women (15, 43, 44) via increased mechanistic target of rapamycin in complex 1 (mTORC1) signaling (45), it also appears that endogenous total testosterone may have an important role in regulating postprandial postexercise MyoPS in women, and to a greater degree than that previously proposed for estradiol. Hence, further investigation is required, especially assessing sexual dimorphism in androgen receptor function and sensitivity, which is thought to drive muscle adaptation (46, 47) and is a step change in how we view the roles of endogenous sex hormones in female muscle metabolism.

To further understand any role of estradiol on MyoPS, we used an AV-V balance approach to comprehensively determine the net balance of each individual AA, and, as phenylalanine is not metabolized across the forearm, AA uptake from MPS and release from MPB in nonexercised tissue. To our knowledge, this AV-V balance technique, which we and others have previously demonstrated to be a robust approach to assess fatty acid, glucose, and AA balance and uptake following various interventions (30, 31, 33, 48), has not previously been used to assess AA balance between phases of the MC. This also gave us the opportunity to observe any differences in the postprandial response to all individual AAs across the MC, which may be affected by other whole-body effects of increased estradiol, such as endometrial growth (49) or altered AA oxidation rates (50). Thus, as typically observed following mixed meal ingestion (0.3 g.kg body mass protein) (31), there was an increase in forearm phenylalanine balance in response to AA ingestion in the present study, underpinned by an increase in muscle AA uptake from MPS and decrease in AA release from MPB, reflecting an anabolic muscle milieu. These MPS data directly agree with our leg MyoPS data, where in a postprandial postexercise state, MyoPS was elevated. However, there was no significant difference in forearm AA balance (of all AAs measured), uptake from MPS (R_d_ of phenylalanine), or release from MPB (R_a_ of phenylalanine) between low and high estradiol concentrations across the MC, which supports our lack of difference in MyoPS at the leg. Moreover, there was no difference in circulating concentration of any AA across phases following AA ingestion, suggesting that estradiol also does not have a measurable effect on any whole-body protein-dependent process. Thus, despite the observed increases in blood flow, insulin, and decreased phenylalanine in the LF phase, we did not observe any major differences in AA metabolism between MC phases, which is in agreement with others (20).

For mechanistic underpinning of any role of estradiol in the MPS response, we measured the expression of genes responsible for the regulation of muscle mass, remodeling, and inflammation during the EF and LF phases. We show for the first time a strikingly different profile in the expression of genes associated with elevated protein synthesis (eg, increased EIF4EBP1, MTOR, RPS6KB1, and SLC38A2, and decreased MSTN expression), and reduced protein breakdown (eg, decreased FBXO32 and TRIM63 expression), with elevated estradiol concentrations during the LF vs EF phase. In agreement with our findings, users of hormone replacement therapy (containing estradiol and progesterone) showed a 68% decrease in expression of the gene encoding myostatin (MSTN), a negative regulator of muscle growth, at rest compared to nonusers (51). However, Smith et al (15) found no difference in postabsorptive MSTN gene expression in postmenopausal women who were supplemented with estradiol, compared to nonsupplemented controls. This contradictory finding may be a result of the estradiol-supplemented group only increasing their circulating estradiol concentration to 422 pmol.L^−1^, which was not much greater than the control group of the study (339 pmol.L^−1^) (15), and well below the concentrations observed in the present study (855 pmol.L^−1^). Alternatively, it could be concluded that progesterone, rather than estradiol, is the anabolic hormone within hormone replacement therapy, which when supplemented in isolation stimulates MPS (15). Moreover, expression of genes associated with the ubiquitin-proteasome pathway, the main pathway responsible for proteolysis within skeletal muscle (52), such as TRIM63, has previously been shown to be increased with estradiol deficiency in postmenopausal women at rest and posteccentric exercise (51). Thus, it would appear that increased estradiol in the LF phase creates a gene expression profile consistent with an increased anabolic environment, despite no change in physiological markers of anabolism, such as increased MyoPS, AA uptake from MPS, and forearm AA balance or decreased AA release from MPB. The reason for this increased anabolic environment is not clear, but gene expression of a key ER present in skeletal muscle, ERα, was trending to be greater in the LF compared to the EF phase. ERα has previously been demonstrated to fluctuate over the course of the MC (18) and is a known transcriptional activator of genes and signaling pathways involved in skeletal muscle protein turnover on binding with estradiol, such as the AKT pathway (10, 13). Indeed, genes encoding several proteins that are negative regulators of muscle mass and known to be inhibited by the AKT pathway were reduced to a greater degree in a postprandial postexercise state during the LF phase, such as MSTN, and FOXO3. Moreover, in ovariectomized female rats, exogenous estradiol has been shown to reduce muscle damage and increase satellite cell activation through ER-mediated mechanisms (12), so it was also interesting to note a greater expression of genes involved in muscle remodeling in the LF phase (CCND1, HSP90AA1, and PAX7), particularly in a postprandial postexercise state (HSPB1, HSPB8, MYOD1, and TGFB1). This would also fit with a robust increase in expression of genes involved in inflammation in the LF phase (NFKB1, NFKBIA, and SOD2) and post exercise (IL18, IL1R1, RELA, and SOD1), given that inflammation appears essential for muscle remodeling and hypertrophy (53). Taking the aforementioned points together, this suggests that there is an environment for overall muscle protein turnover to be greater during the LF phase, where estradiol is elevated. It is intriguing, therefore, why resistance exercise or provision of AA did not influence R_d_, R_a_, or MyoPS during the LF phase, particularly as testosterone and insulin were also elevated, albeit moderately, and are known to affect protein synthesis and breakdown signaling pathways and processes (45, 54, 55). Perhaps a larger, whole-body, resistance exercise stimulus or a more sustained exposure to this hormonal environment is required to observe any physiological effect.

Some limitations are worth noting. A unilateral, dominant-leg, exercise model may have limited the working tissues need for protein synthesis, thereby decreasing the ability to pick up differences. Likewise, there was a trend for workload to be 6% higher in the LF phase. However, we and others have used this approach on several occasions and demonstrated an FSR response similar to when bilateral (56) or whole-body (34) exercise is performed in our laboratory. Related to this, despite habitually consuming 1.2 g·kg^−1^ BM·day^−1^ protein (considered optional for skeletal muscle adaptation to training or exercise performance (57)), participants were fasted on arrival and likely in an energy deficit, even following AA drink consumption. Hence, to strengthen the practical relevance of these findings future work should assess MyoPS when in (positive) energy balance and performing more ecologically valid resistance exercise. Furthermore, we did not assess participants’ sleep, which is known to be affected by MC phase (58) and even one night of sleep deprivation is known to blunt MPS responses (59). Finally, we have measured only gene expression but due to various posttranscriptional modifications, changes in mRNA expression do not always necessarily reflect active protein content, hence measurements of protein expression, particularly during a training study, may provide more insight.

In conclusion, for the first time we demonstrate that MyoPS did not differ with low and high estradiol concentrations during the EF and LF phases of the MC, validated with the gold-standard 3-step method, in a postabsorptive and postprandial postexercise state. Furthermore, MyoPS did not correlate with fluctuating estradiol concentrations across the MC, suggesting estradiol is not important for the regulation of muscle mass in women. This is despite a clear and robust gene expression profile consistent with muscle growth during the LF phase. In contrast, we report for the first time that small variations in circulating testosterone correlated with MyoPS across the MC, demonstrating the complexity of trying to isolate the effect of a single sex hormone on MyoPS in vivo. Further research elucidating the role of sex hormones in skeletal muscle, and aiming to optimize MPS, hypertrophy, adaptation, and recovery in women, is clearly warranted.

## Data Availability

The data sets generated during and analyzed during the current study are not publicly available but are available from the corresponding author on reasonable request.

## Clinical Trial Information

Clinical Trials registration number NCT05178732 (registered December 7, 2021).

## Abbreviations

AA: amino acid
AKT: protein kinase B
ANOVA: analysis of variance
AV-V: arterialized venous-deep venous
BCAA: branched-chain amino acids
BMI: body mass index
EAA: essential amino acids
EF: early follicular
ER: estrogen receptor
FSH: follicle-stimulating hormone
FSR: fractional synthetic rate
GAE: gallic acid equivalent
GC-MS: gas chromatography-mass spectrometry
LF: late follicular
LH: luteinizing hormone
MC: menstrual cycle
ML: mid-luteal
MPB: muscle protein breakdown
MPE: mole percent excess
MPS: muscle protein synthesis
mRNA: messenger RNA
MyoPS: myofibrillar protein synthesis
R_a_: rate of appearance
R_d_: rate of disappearance
RM: repetition maximum
SHBG: sex hormone–binding globulin
TAA: total amino acids
